# Interaction effect of response medium and working memory capacity on creative idea generation

**DOI:** 10.3389/fpsyg.2015.01582

**Published:** 2015-10-13

**Authors:** Ning Hao, Huan Yuan, Rui Cheng, Qing Wang, Mark A. Runco

**Affiliations:** ^1^School of Psychology and Cognitive Science, East China Normal UniversityShanghai, China; ^2^American Institute for Behavioral Research and TechnologySan Diego, CA, USA

**Keywords:** creativity, creative idea generation, divergent thinking, working memory capacity, response medium

## Abstract

This study aimed to examine the interaction effect of response medium (i.e., write down ideas and orally report ideas) and working memory capacity (WMC) on creative idea generation. Participants (*N* = 90) with higher or lower WMC were asked to solve Alternative Uses Task (AUT) problems in the condition of writing down or speaking out ideas. The results showed that fluency of AUT performance was higher in the writing than in the speaking condition. Additionally, participants with higher WMC performed better on AUT fluency than those with lower WMC in the writing condition, while they showed no difference in the speaking condition. Moreover, level of cognitive demand fully mediated the effect of response medium on AUT fluency. Theoretically, these findings indicated the importance of WMC in creative idea generation, which supported the controlled-attention theory of creativity. Practical implications and future directions were discussed.

## Introduction

Creativity is defined as the ability to produce work that is novel (original, unique) and useful (Sternberg and Lubart, 1996; Runco and Jaeger, 2012). Idea generation is one of the fundamental processes of creative thinking (Runco, 2003; Sowden et al., 2015), which has been emphasized in various models of creativity. The blind variation and selective retention (BVSR) theory of creativity (Campbell, 1960), for example, is a two-step model in essence, which emphasizes the totally random or “blind” variation, followed by selection of better ideas and their retention by the culture. The Darwinian theory of creativity (Simonton, 1999, 2007, 2010, 2012, 2013), which has its roots in the BVSR theory, includes a similar two-step process in which the generation of ideas is followed by evaluation of those ideas. The Genoplore model (Finke et al., 1992) suggests that creative thinking consists of idea generation and idea exploration. It has been demonstrated that participants' performance on creative idea generation is a reliable predictor of actual, real-world creative performance (Runco and Acar, 2012).

Divergent thinking (DT) tasks are commonly used to measure creative idea generation, such as the Alternative Uses task (AUT) (Guilford, 1967), the Instances task (Wallach and Kogan, 1965), and so on. While working on the DT tasks, respondents are usually encouraged to generate as many unusual or original ideas as possible. In plenty of studies using DT tasks, participants were asked to write down their generated ideas briefly by paper and pencil (Gilhooly et al., 2007; Leung et al., 2012; Beaty et al., 2014; Radel et al., 2015). Yet in many other studies, participants were required to speak out ideas one by one, and their oral responses were recorded by a voice recorder (Fink et al., 2007, 2011; Hao et al., 2014, 2015; Oppezzo and Schwartz, 2014). Here arises an interesting question: which medium, writing down ideas or speaking out ideas, is better for creative idea generation? Notably, this question is not just a methodological issue but also has its implication for daily creative problem solving. For example, when people ponder on problems, would writing down ideas or speaking them out with a voice recorder facilitate the generation of creative solutions?

Creative idea generation is, according to the controlled-attention theory of creative cognition (Beaty et al., 2014; Jauk et al., 2014), a top-down process that involves many control processes (Runco, 1994). These include inhibiting interference from external unrelated stimuli (Fink et al., 2009, 2010; Benedek et al., 2011, 2014a), inhibiting dominant but not novel responses (Nusbaum and Silvia, 2011; Beaty and Silvia, 2012; Silvia and Beaty, 2012), conducting directed search and retrieval processes (Beaty and Silvia, 2013; Silvia et al., 2013), and evaluating and refining initial ideas (Finke et al., 1992; Runco and Smith, 1992; Gabora, 2005; Vartanian, 2011). Following these research lines, working memory capacity (WMC), the ability or construct to hold the current task-related information as well as to inhibit task-irrelevant intervention (Baddeley, 2003), has been suggested to affect creative idea generation. This proposal has been supported by solid findings that WMC is positively correlated with performance on creative idea generation (Oberauer et al., 2008; De Dreu et al., 2012; Lee and Therriault, 2013).

Notably, taxing working memory resources by one activity would reduce the resources to be used by another concurrent activity (Van Dillen and Koole, 2007). Hence, writing down ideas and speaking out ideas, which are supposed to have different cognitive demands and consume different amount of working memory resources, would have different effects on creative idea generation. Conceivably, while speaking out ideas one by one, people have to hold the current idea; meanwhile, they have to search the memory and compare this idea with the ideas just generated (i.e., old ideas) in case they report it repeatedly. These cognitive processes call for a lot of working memory resources. In contrast, though writing down ideas also involves comparison of the current idea and old ideas, people do not need to hold the idea and search the memory because all old ideas have been written down on the paper. Consequently, writing down ideas might have lower cognitive demand and consume less working memory resources compared with speaking out ideas, which benefits creative idea generation as a result.

Individual differences of WMC should be considered when exploring the effect of response medium (i.e., writing down or speaking out) on creative idea generation. It is demonstrated that participants with higher WMC performed better on fluency and originality of idea generation (De Dreu et al., 2012). What we hypothesize here is that there might be an interaction effect of response medium and WMC on creative idea generation. Specifically, in the context of writing down ideas, a small quantity of working memory resources is consumed. Thus, people with higher WMC would perform better on creative idea generation than those with lower WMC. In the context of speaking out ideas one by one, however, the response medium calls for much more working memory resources, thus people with higher WMC might have no vantage of available WMC to perform better than those with lower WMC. Therefore, the aims of the present study are not only to investigate the effect of response medium on creative idea generation, but also to explore the interaction effect of response medium and individual WMC level on creative idea generation.

In this study, participants were asked to work on a DT task in the writing or speaking condition. Their WMC was then measured by means of a WMC task. To check whether different response mediums might elicit different emotional states, which have complex effects on creative cognition (Baas et al., 2008; De Dreu et al., 2008), participants' emotional valence and arousal were measured before and after the experiment. Moreover, cognitive demands elicited by writing down and speaking out ideas were also measured in order to test whether different response mediums would influence creative ideation because of their different cognitive demands.

The main hypotheses were as follows. First, participants would perform better on creative idea generation in the writing than in the speaking condition, given that writing down ideas might have lower cognitive demand compared with speaking out ideas. Second, participants with higher WMC would perform better on idea generation than those with lower WMC. Third, there might be an interaction effect of response medium and WMC on idea generation. Specifically, because of high cognitive demand of speaking out ideas, participants with higher or lower WMC might show no difference on ideation performance in this case; however, in the writing condition where writing down ideas had low cognitive demand, participants with higher WMC would perform better than those with lower WMC. Fourth, cognitive demand of response medium might mediate the effect of response medium on creative ideation.

## Methods

### Participants

Ninety right-handed college students (32 male and 58 female; *M* = 21.49 years, *SD* = 1.20, Range: 19–24 years), who majored in various academic disciplines, participated individually in this study. They were all native Chinese speakers. They were randomly assigned to one of the two experimental conditions: writing down ideas or speaking out ideas. The gender ratio was same in the two groups. The *t*-test revealed that the mean age of the two groups did not differ from each other [*t*_(88)_ = 1.23, *p* = 0.22], nor did the mean WMC levels [*t*_(88)_ = 1.21, *p* = 0.23] (see Table 1). All participants were gave written informed consent and were paid about 4 dollars for their participation. The protocol of the experiment was approved by the Institutional Ethics Committee at East China Normal University.

**Table 1:** **Descriptive statistics of individuals in the speaking and writing groups**.

**Group**	**Number of Female/Male**	**Age (*M* ±*SD*)**	**WMC (*M* ±*SD*)**
Speaking	29/16	21.33 ± 1.24	3.69 ± 1.69
Writing	29/16	21.64 ± 1.15	4.13 ± 1.80

Individuals' WMC was measured by the Reading Span Task.

### Experimental tasks

The Alternative Uses Task (AUT) (Guilford, 1967) was used as the target task. It required respondents to generate as many unusual or original uses as possible for common objects, such as a paperclip (“making a ring,” “cleaning fingernails”). The AUT is a well-established test of creative potential (Guilford, 1967; Runco, 1991, 1999; Runco and Mraz, 1992). Performance on this task has been demonstrated to be a reliable predictor of actual, real-world creative performance (Runco and Acar, 2012).

The Reading Span Task (Daneman and Carpenter, 1980) was adopted to measure participants' WMC. Operationally defined as the number of items that can be recalled during complex span tasks, WMC mainly fulfills two classes of function: to actively maintain information, and to discriminate task-relevant and task-irrelevant information (Unsworth and Engle, 2007). As the Reading Span Task requires participants to judge whether or not the sentences presented on the screen are reasonable while holding the last words of the sentences in their mind, it is regarded as a typical task to measure WMC (Daneman and Carpenter, 1980; Baddeley, 2003). Specifically, following a fixation lasting for 1000 ms, a sentence appeared on the screen. Participants needed to make the judgment by pressing key 1 or 0 (1 = true; 0 = false) while memorizing the last word in 4000 ms. Or else, a warning message would appear on the screen, suggesting that no response was detected. When a series of sentences were finished, participants were required to enter the words sequentially into the computer with a semicolon following each word. The task consisted of six levels from level 2 to level 7 (i.e., memorizing 2–7 words). Each level contained three groups of sentences. The number of sentences in each group was in line with the level. For example, a group contained two sentences if the level was 2. Only when participants entered all the words of each group correctly could they make a point. Participants were permitted to step up to a higher level if they got points of 2 or above. If they failed, the task ended. The last fully completed level was the participant's performance score. For example, if a participant stopped at level 5, it means he or she completed the 3 groups of level 4 successfully, and would achieve the score of “4” for Reading Span Task.

### Experimental procedure

Participants were informed that they would solve two AUT problems (i.e., brick and chopstick), and then complete the Reading Span Task. In the instruction about how to solve the AUT problems, participants were encouraged to try their best to produce ideas that would be thought of by no one else, as suggested by Harrington (1975), Runco and Pritzker (1999), and Torrance (1995).

Participants solved these two AUT problems in 20 min (i.e., 10 min per problem), with a 1-min break between the two problems. In the writing condition, participants were asked to write down their ideas briefly by paper and pencil. In the speaking condition, participants were asked to report their ideas orally. The oral responses for the AUT problems were recorded by a voice recorder and transcribed afterwards for further analysis.

### Pre- and post-experimental tests

Prior to the experiment and immediately after the experiment, participants self-rated the valence and arousal levels of their emotional states by means of the Self-Assessment Manikin (Bradley and Lang, 1994), in which they selected one of nine ratings (valence: 1 = very unpleasant, 9 = very pleasant; arousal: 1 = not exciting at all, 9 = very exciting) illustrated by five cartoon figures and the points between any two figures.

Moreover, after participants completed the experiment, their effortlessness involved in writing down or speaking out ideas was measured by asking: “How effortful was it to write down (or speak out) ideas” on a scale ranging from 1 (not at all effortful) to 5 (very effortful). Such a technique (i.e., self-reported mental effort ratings) is widely used to measure the level of cognitive demand of a task. It has been proven to be most sensitive to reflect the cognitive demand of intrinsic processing elicited by the task relative to another two techniques for measuring cognitive demand (i.e., response time to a secondary task during task performance, and difficulty ratings of task; Paas et al., 2003; Ayres, 2006; DeLeeuw and Mayer, 2008).

### AUT performance assessment

Participants' performance on the AUT problems was measured on the scores of fluency and originality (Guilford, 1967; Runco, 1991; Runco and Pritzker, 1999). Fluency scores were based on the total number of ideas of the given AUT problems. Originality scores were based on statistically infrequent responses. To this end, the ideas generated by all participants were collected into a comprehensive lexicon. Synonyms were identified and ideas collapsed accordingly. If a response was statistically infrequent (i.e., if 5% or less participants in the sample gave the response), it was given a score of “1.” All other responses received scores of “0,” regardless of how often they appeared. Following this scoring procedure, two trained raters independently assessed the originality of the two AUT problems for every participant. The inter-rater agreement (ICCs = 0.96) was satisfactory. The internal consistency of the fluency in solving these two problems was satisfactory (Cronbach's alpha coefficient was 0.80), as was that of the originality (alpha was 0.70). Finally, the fluency or originality scores in solving two problems were averaged for every participant.

## Results

### Effects of response medium and WMC on AUT performance

According to the performance on the Reading Span Task, participants were split into higher WMC group (level 5, 6, and 7; 42 participants, 18 male and 24 female) and lower WMC group (level 2, 3, and 4; 48 participants, 14 male and 34 female).

**Figure 1:**
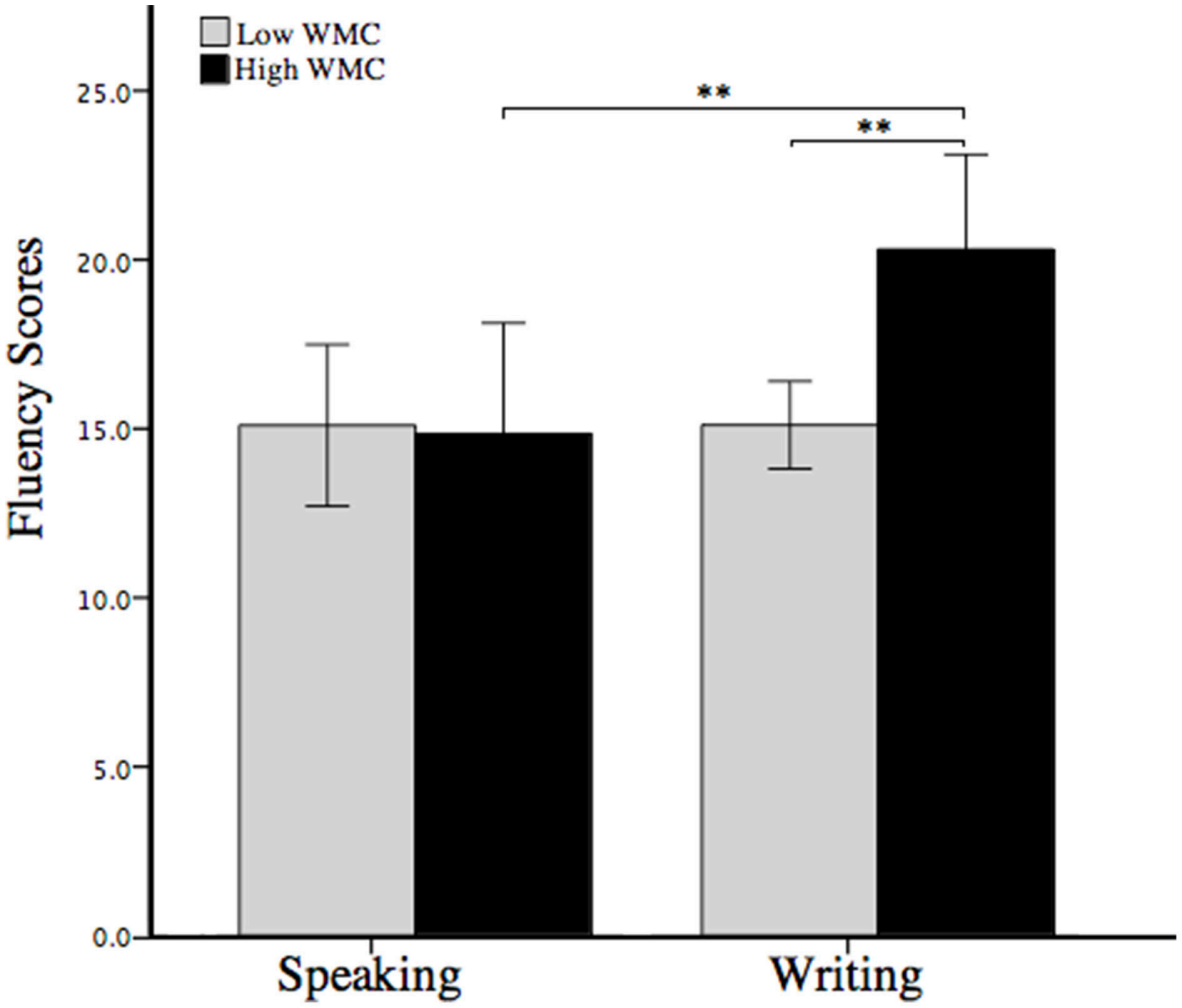
**Alternative Uses Task (AUT) fluency scores of participants with higher or lower working memory capacity (WMC) in the speaking and writing conditions**. Error bars indicate standard errors of the mean. ^**^p < 0.01.

A Two-Way ANOVA with response medium (writing vs. speaking) and WMC (higher vs. lower) as between-subject factors was performed on the fluency scores. As shown in Figure 1, there was a significant main effect of WMC, *F*_(1, 86)_ = 4.21, *p* < 0.05, $\eta_{p}^{2}=0.05$. Overall, participants with higher WMC generated more ideas (*M* = 17.70, *SD* = 7.14) than those with lower WMC did (*M* = 15.10, *SD* = 6.62). Also, there was a significant main effect of response medium, *F*_(1, 86)_ = 5.14, *p* < 0.05, $\eta_{p}^{2}=0.06$. That is, participants produced more ideas in the writing condition (*M* = 17.64, *SD* = 5.53) than in the speaking condition (*M* = 15.00, *SD* = 6.28). More interestingly, a significant interaction effect of response medium and WMC was observed, *F*_(1, 86)_ = 5.11, *p* < 0.05, $\eta_{p}^{2}=0.06$. Specifically, in the writing condition, participants with higher WMC generated more ideas (*M* = 20.30, *SD* = 6.35) than those with lower WMC (*M* = 15.11, *SD* = 3.00), *t*_(43)_ = 3.53, *p* < 0.01, Cohen's *d* = 1.05. However, in the speaking condition, the fluency scores showed no difference between participants with higher or lower WMC (*M* = 14.85, *SD* = 7.00; vs. *M* = 15.10, *SD* = 5.79), *t*_(43)_ = −0.13, *p* = 0.9.

**Figure 2:**
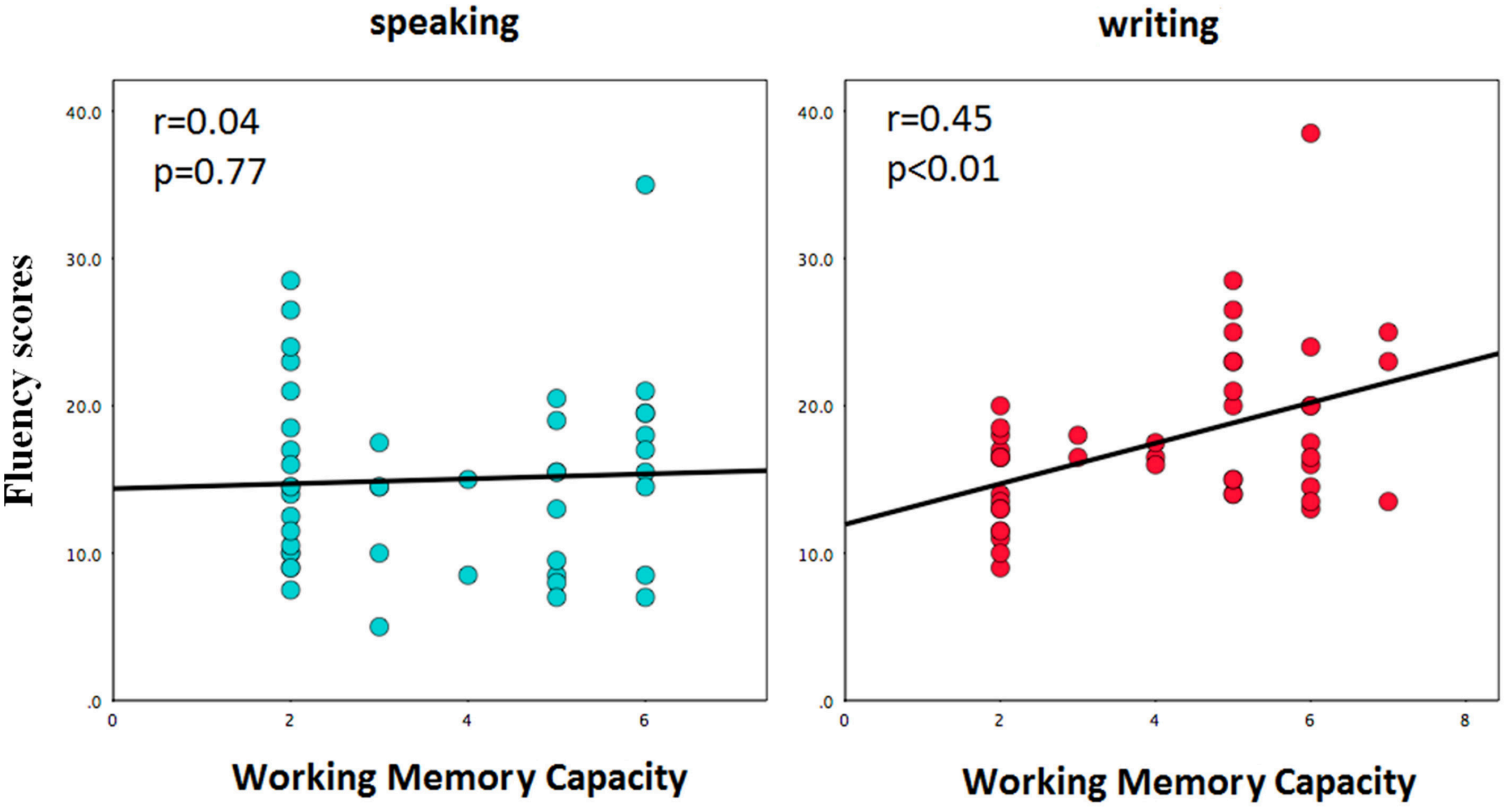
**Correlations between working memory capacity (WMC) and fluency scores in the speaking and writing conditions**.

To further investigate the interaction effect of response medium and WMC, we took participant's WMC level as a continuous variable, and conducted a univariate ANOVA with response medium as the between-subject factor and WMC as a covariate. The results showed that the interaction effect of response medium and WMC on fluency was also significant, *F*_(2, 87)_ = 6.33, *p* < 0.01, $\eta_{p}^{2}=0.13$. In the speaking condition, fluency scores were not correlated with WMC; while in the writing condition, the higher participants' WMC was, the more ideas they generated (*r* = 0.45, *p* < 0.01) (see Figure 2).

For the originality scores, there was no main effect of response medium or WMC. Interaction effect of these two factors was not significant either.

### Mediation effect of cognitive demand between response medium and AUT fluency

Cognitive demand of response mediums was measured by the self-reported mental effort ratings (Paas et al., 2003; Ayres, 2006; DeLeeuw and Mayer, 2008). Participants' self-rated effortfulness of speaking out and writing down ideas was compared. It was found that effortfulness in the speaking condition (*M* = 3.33, *SD* = 1.15) was higher than that in the writing condition (*M* = 2.07, *SD* = 0.92), *t*_(88)_ = 5.79, *p* < 0.001, Cohen's *d* = 1.21.

**Figure 3:**
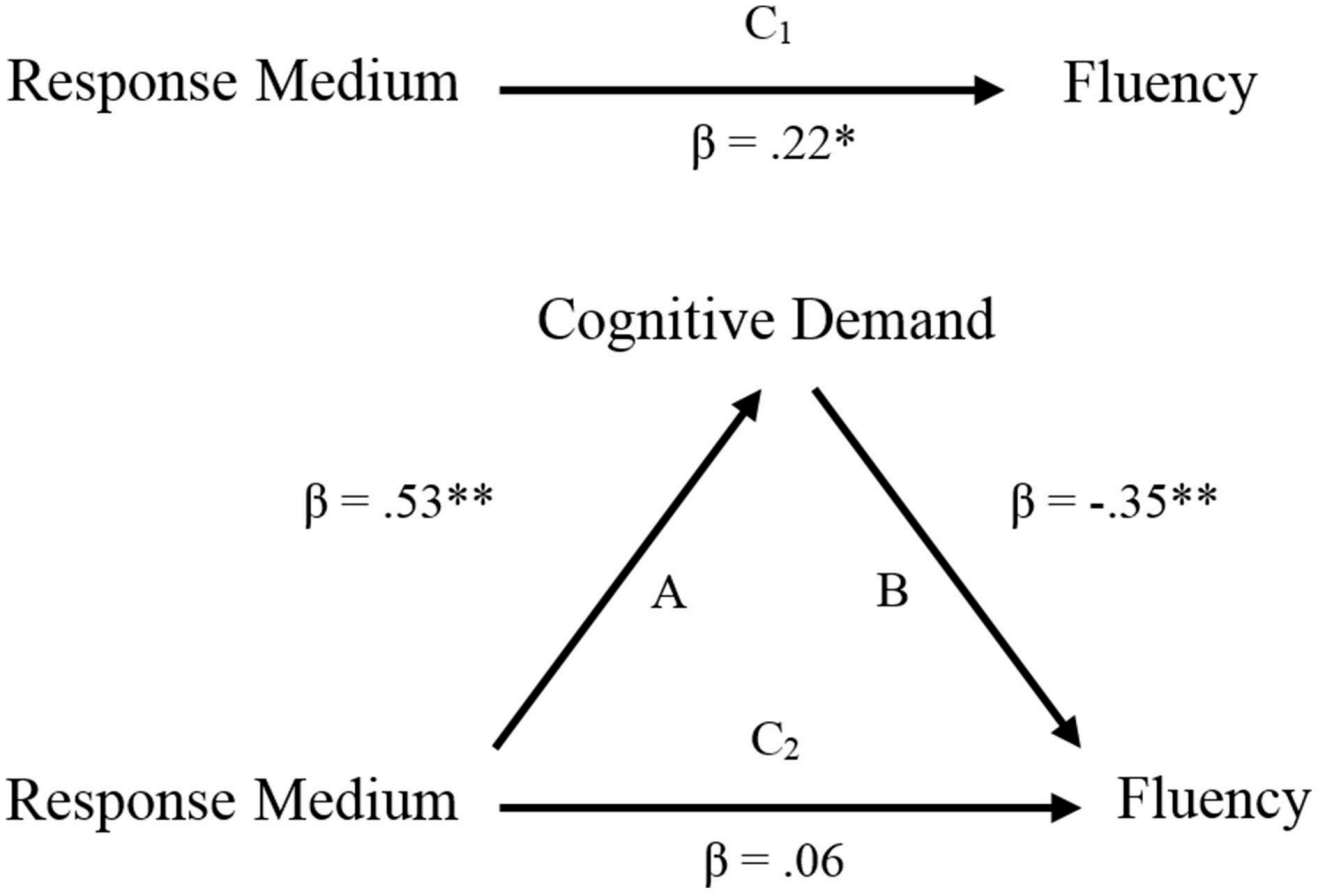
**Mediation effect of cognitive demand between response medium and AUT fluency**. ^*^p < 0.05, ^**^p < 0.01.

Based on the method developed by Baron and Kenny (1986), the mediation effect of cognitive demand on the relation between response medium and AUT fluency was examined with three linear regressions. All variables were centered in order to avoid multicollinearity with the interaction term. First, the outcome variable (i.e., fluency) was regressed on the predictor (i.e., response medium). The result (Path C_1_) was found to be significant (β = 0.22, *p* < 0.05). Second, the relationship (Path A) between the predictor and the mediator variable (i.e., cognitive demand) was significant (β = 0.53, *p* < 0.01), as was path B between the mediator variable and the outcome variable (β = −0.35, *p* < 0.01). Finally, fluency was regressed on both cognitive demand and response medium. The result turned out that prediction effect of response medium (Path C_2_) became insignificant (β = 0.06, *p* = 0.61) (see Figure 3). These results indicated that cognitive demand fully mediated the relation between response medium and AUT fluency.

### Emotions in the writing and speaking conditions

Levels of valence and arousal of emotional states in pre- and post-experimental epochs in the speaking and writing conditions were shown in Table 2. An ANOVA for repeated measures with epoch (pre- vs. post-experimental) as a within-subject factor, and response medium as a between-subject factor, was conducted on the valence levels. The results showed that there was a significant main effect of epoch, *F*_(1, 88)_ = 24.43, *p* < 0.01, $\eta_{p}^{2}=0.22$, but there was neither main effect of response medium nor interaction effect of response medium and epoch. Another ANOVA for repeated measures revealed that epoch had a significant main effect on the arousal levels, *F*_(1, 88)_ = 9.89, *p* < 0.01, $\eta_{p}^{2}=0.10$, but response medium did not. Interaction effect of response medium and epoch was not statistically significant either.

**Table 2:** **Levels of valence and arousal of emotional states in pre- and post-experimental epochs under the speaking and writing conditions (*M* ± *SD*)**.

**Emotion**	**Speaking**		**Writing**	
	**Pre**	**Post**	**Pre**	**Post**
Valence	5.47 ± 1.56	4.71 ± 1.58	6.20 ± 1.42	5.02 ± 1.97
Arousal	4.24 ± 1.87	5.00 ± 2.06	4.22 ± 1.99	5.20 ± 2.02

To further investigate whether emotional states might confound the effect of response medium on AUT fluency, pre- and post-experimental valence and arousal levels were entered into the ANOVA model as a covariate successively. The results revealed that none of these four variables diminished the main effect of response medium or interaction effects of response medium and WMC on AUT fluency.

## Discussion

The present study aimed to investigate which response medium, writing or speaking, was better for creative idea generation; as well to explore how response medium and WMC exerted an interaction effect on creative idea generation. It was revealed that participants in the writing condition generated more ideas than those in the speaking condition. And, there was an interaction effect of response medium and WMC on AUT fluency. Participants with higher WMC produced more ideas than those with lower WMC in the writing condition, but such difference was not observed in the speaking condition. More interestingly, it was demonstrated that level of cognitive demand mediated the effect of response medium on AUT fluency. To our knowledge, the findings in this study make an innovative and significant contribution to the research on creativity.

Compared with speaking out ideas, writing down ideas helped people generate more responses (see Figure 1). This finding was consistent with the first hypothesis of the study. In the writing condition, the generated ideas had been written down on the paper, so participants did not have to employ many working memory resources to hold the current idea and search memory to compare it with old ideas. Therefore, more WMC could be spared to the cognitive processes involved in creative idea generation, such as inhibiting interference of external unrelated stimuli (Fink et al., 2009, 2010; Benedek et al., 2011, 2014b), overriding the generation of most accessible but common ideas (Nusbaum and Silvia, 2011; Beaty and Silvia, 2012; Silvia and Beaty, 2012), combining concepts retrieved from long-term memory (Mednick, 1962; Paulus and Brown, 2007), and so on. However, in the speaking condition, participants had to mentally compare every newly generated idea with the old ideas in the memory, which demanded a lot of working memory resources, particularly in a long period of task performance (i.e., 10 min) in this study. Notably, participants' self-rated effortfulness was higher for speaking out ideas than writing down ideas. This meant speaking out ideas elicited higher cognitive demand (Paas et al., 2003; Ayres, 2006; DeLeeuw and Mayer, 2008). As a result, less WMC was used in cognitive processes involved in idea generation in the speaking condition, which might then impair AUT fluency.

The explanation above was strongly supported by the results of mediation effect analysis (see Figure 3). It was demonstrated that cognitive demand fully mediated the effect of response medium on AUT fluency. In addition, higher cognitive demand was associated to lower fluency scores. These findings indicated that writing down or speaking out ideas, seemly just different in the facet of response medium, did affect creative idea generation through the cognitive demand they elicited.

The current study revealed that higher WMC was conducive to creative idea generation, similar to the findings of previous studies (Oberauer et al., 2008; De Dreu et al., 2012; Lee and Therriault, 2013). More importantly, this study found an interaction effect between response medium and WMC on AUT fluency (see Figures 1, 2). Specifically, only in the writing condition did participants with higher WMC perform better on AUT fluency than lower WMC; while these two groups of participants showed no difference in the speaking condition. These might because speaking out ideas one by one elicited high cognitive demand and occupied a large amount of working memory resources, which was a challenging task for participants with either higher or lower WMC. Thus, the AUT fluency showed no difference between these two groups of participants. By contrast, writing down was a relatively less resource-consuming way to report ideas. Consequently, in the writing condition, participants with higher WMC spared more cognitive control resources and then generated more ideas than those with lower WMC.

Valance and arousal of emotion showed no difference between the writing and speaking conditions, no matter in the pre- or post-experimental epoch (see Table 2). In addition, emotional states of the two epochs did not confound the effect of response medium and its interaction with WMC on AUT fluency. These findings refuted the possibility that the effects of writing down and speaking out ideas on creative idea generation could be resulted from the different emotional states induced by different response mediums.

There has been a theoretical debate on whether creative thinking relied mainly on implicit or explicit processing. Some researchers argued that implicit associative process, which was beneficial for accessing distantly related information (Dijksterhuis and Meurs, 2006), could spark creative ideas or solutions. On the contrary, other researchers claimed that explicit executive process, such as overriding the habitual responses (Gilhooly et al., 2007; De Dreu et al., 2012), would be indispensable for creative thinking. It is well acknowledged that explicit processing is highly correlated with WMC, whereas implicit processing is not (Kane et al., 2001; Evans, 2008). Thus, to test the role of WMC in creative idea generation would provide evidence to distinguish the implicit from the explicit account. Many previous studies showed that WMC was not related to creativity performance. For instance, Furley and Memmert (2015) revealed that domain-general WMC was not associated with creativity in a soccer-specific creativity task. DeYoung et al. (2008) found that WMC (measured by a self-ordered pointing task) was not associated with DT performance. Takeuchi et al. (2011) reported that the reduced task-inducted deactivation (TID) in the precuneus, which indicated the inefficient reallocation of working memory (WM) resources, was associated with better DT performance. These studies seemed to support the implicit account of creativity. However, there were also plenty of studies showing that WMC was positively correlated with performance on creative idea generation (Oberauer et al., 2008; De Dreu et al., 2012; Lee and Therriault, 2013). In line with these studies, our findings indicated the importance of WMC in creative idea generation. Theoretically, the present study supported the explicit account of creativity. Notably, some recent researchers proposed that both implicit and explicit processing were simultaneously involved in most creativity tasks (Hélie and Sun, 2010; Allen and Thomas, 2011; Sowden et al., 2015). Future research should explore the underlying mechanism on how implicit and explicit processing may interact to generate creative ideas.

It must be pointed out that speaking out ideas is a priority to writing down ideas in some given experimental contexts. First, with the application of neuroscience methods (e.g., fMRI, EEG) in psychology, experimental tasks by means of paper-and-pencil become inappropriate. For example, participants are allowed little motion of body even their heads in fMRI experiments. In this case, writing down ideas with hand is not allowed; orally reporting ideas and recording them with a voice recorder is a good alternative (Fink et al., 2007). Second, in the studies where participants are allowed a very short period of time (i.e., 4, 10 s) to give responses, speaking out ideas excels writing down ideas, given that writing is more time consuming than speaking. In addition, it should be noted that the originality of idea generation showed no difference between two response conditions in the current study. It may imply that both writing down and speaking out ideas are appropriate ways in the studies that emphasize originality instead of fluency of idea generation.

There are three limitations of this study. First, there might be a problem of reliability in using only one task (i.e., AUT). Further research should adopt more than one verbal DT tasks (e.g., Instances task; Wallach and Kogan, 1965) to justify the reliability of our findings. Second, this study adopted a 10-min paradigm of performing AU task. Notably, time affects creativity (Runco, 1999). As the time passes by, fluency of idea generation decreases and originality of idea generation increases (Parnes, 1961; Ward, 1969; Beaty and Silvia, 2012). Further research should manipulate different periods of time (e.g., 3, 5 min) to testify whether the interaction effect of response medium and WMC on idea generation is solid in various periods of task performance. Third, this study did not measure individuals' intelligence. As participants were randomly assigned into the speaking or writing condition, it is reasonable to propose that intelligence of the two groups was statistically equal. However, given that intelligence is related to working memory (Süßet al., 2002; Oberauer et al., 2008) and creativity (Jauk et al., 2013), future study should involve intelligence as a variable to explore whether it affects the interaction effect of WMC and response medium on creativity.

### Conflict of interest statement

The authors declare that the research was conducted in the absence of any commercial or financial relationships that could be construed as a potential conflict of interest.
